# *Staphylococcus aureus, Escherichia coli*, and *Klebsiella* spp. prevalence in bulk tank milk of Colombian herds and associated milking practices

**DOI:** 10.14202/vetworld.2023.869-881

**Published:** 2023-04-26

**Authors:** Ángela Sofía Ágredo-Campos, Jorge A. Fernández-Silva, Nicolás F. Ramírez-Vásquez

**Affiliations:** Centauro, Escuela de Medicina Veterinaria, Facultad de Ciencias Agrarias, Universidad de Antioquia UdeA, Calle 70 No. 52-2, Medellín, Colombia

**Keywords:** bovine mastitis, bulk tank milk, milking, raw milk pathogens, somatic cell count

## Abstract

**Background and Aim:**

Bovine mastitis is one of the most costly and prevalent diseases in dairy herds, which can be prevented and controlled through proper milking practices, diagnosis, and elimination of chronic animals, among others. Contagious pathogens such as *Staphylococcus aureus* and environmental pathogens such as *Escherichia coli* and *Klebsiella* spp. can affect cows and milk for human consumption, generating a public health risk. This study aimed to estimate the prevalence of herds with somatic cell count (SCC) ≥200,000 cells/mL, *S. aureus*, *E. coli*, and *Klebsiella* spp., in bulk tank milk (BTM) and its associated risk factors in Colombian dairy cow herds.

**Materials and Methods:**

A cross-sectional probabilistic study was carried out in 150 dairy herds located in the north of the Antioquia province. A single visit per herd was conducted, during which three BTM samples were aseptically collected. General data and milking practices were collected through an epidemiological survey applied in each herd.

**Results:**

The prevalence of *S. aureus*, *E. coli*, and *Klebsiella* spp. were 14% (21/150), 2% (3/150), and 8% (12/150), respectively. Moreover, 95% of the herds presented an SCC of ≥200,000 cells/mL. Practices such as in-paddock milking, change of milker during the last month, use of disposable gloves, and hand disinfection were associated with increased prevalence of *S. aureus*, whereas proper dipping was a protective factor. Proper washing and disinfection of the milking machine, use of chlorinated disinfectants for hand hygiene, and use of disposable gloves decreased the prevalence of *E. coli* and *Klebsiella* spp. Bulk tank SCC increased in herds with 30–60 milking cows, herds with >60 milking cows, and herds with a change of milker during the last month. Hand disinfection and dipping decreased the SCC.

**Conclusion:**

*Staphylococcus aureus*, *E. coli*, and *Klebsiella* spp. were prevalent in BTM from dairy cow herds. The risk of *S. aureus* isolation was higher in herds with an in-paddock milking system. The risk of *E. coli* and *Klebsiella* spp. isolation were higher in herds with >60 milking cows, with a change of milker during the last month. Processes such as avoiding changing the milker and greater control in medium and large herds could improve the SCC in BTM.

## Introduction

Bovine mastitis is one of the most important diseases of the udder tissue, affects specialized dairy farming, and is the most common and costly disease in dairy herds worldwide [1–3]. Economic losses are due to a decreased milk yield, premature culling of animals, prolonged costly antibiotic treatments, and cost of control and prevention measures [4]. A study conducted in Colombia estimated losses of U$692 per herd because of subclinical mastitis (SM) [5]. Subclinical mastitis decreases milk quality by increasing the somatic cell count (SCC) and colony-forming units, which make marketed raw milk a less competitive product at an industrial level because of decreased raw milk conversion in cheese and dairy products [6].

The etiopathology of bovine mastitis is complex and information on the prevalence and distribution of mastitis-causing bacteria [7, 8], as well as the identification of risk factors [9], is crucial for its control and/or prevention. Bulk tank milk (BTM) sample testing is an effective approach to assessing milk quality at the herd-level and is useful for detecting and identifying the causative pathogens in cows clinically affected by mastitis [10]. Isolation of mastitis pathogens from BTM indicates that contamination or intramammary infection is present in one or more cows on the farm [8, 11]. The most frequently isolated pathogen is *Staphylococcus* *aureus*, which is the prime etiological agent of contagious bovine mastitis and is closely linked to milking hygiene, as well as udder and leg hygiene [1, 12]. Intramammary infections in dairy cattle caused by *S. aureus* are difficult to treat and particularly challenging because of their tendency to become chronic and recur [13] and their ability to invade epithelial cells and form a biofilm, and polysaccharide capsules appear to be most commonly associated intracellular persistence [14]. In addition, *S. aureus* strains have the potential to express several enterotoxins in the case of foodborne intoxication in humans [15]. *Staphylococcus aureus* food contamination by food handlers is considered the most common source of staphylococcal food poisoning; however, outbreaks have also been associated with the consumption of raw milk or raw milk cheese derived from dairy animals with mastitis [16]. In addition, several bacterial species, including *Escherichia coli* and *Klebsiella* spp., are known as the main bovine mastitis pathogens associated with environmental mastitis [17]. These main coliform bacteria of concern in BTM include organisms that are pathogenic to humans and animals and those that lower the quality of milk.

In Colombia, several non-probabilistic studies have reported the presence of contagious and environmental pathogens as a cause of intramammary infections in dairy cattle [18–21]. Some of these studies have shown a higher prevalence of *S. aureus* in small dairy cattle herds [22]. In addition, risk factors associated with the presentation of mastitis caused by contagious pathogens have been detected inside the herds, such as the absence of hand washing, manual milking, mechanical milking in the paddocks, lack of pre-dipping and post-dipping, inadequate dilution of dipping solutions, Holstein animals, and animals with more than one calving [21, 23, 24]. Reyes *et al*. [25] reported that BTM SCC increased in herds with manual milking and fore-stripping and dipping failures, whereas the lowest SCC was noted in herds with a milking machine and pre-dipping and dipping at the end of milking. Nevertheless, probabilistic and updated studies on the presence of mastitis-related contagious and environmental pathogens and herd-level risk factors in Latin American dairy cow herds are scarce in general. Therefore, generating knowledge about these issues under tropical conditions is important to improve milk quality and yield and consequently obtain acceptable risk levels for the consumer.

Considering the above, this study aimed to estimate the prevalence of herds with SCC ≥200,000 cells/mL, *S. aureus*, *E. coli*, and *Klebsiella* spp., in BTM and associated risk factors in Colombian dairy cow herds.

## Materials and Methods

### Ethical approval and informed consent

This study was approved by the Ethics Committee for Experimentation with Animals of the Universidad de Antioquia, Colombia (Act No. 110 of May 17, 2017). Farmers provided informed consent before data collection.

### Study period, location, and population

The study was conducted from August 2019 to January 2020 in the municipalities of Santa Rosa de Osos, Entrerríos, and San Pedro de los Milagros, which are recognized as having the highest milk production in the north of Antioquia, Colombia. The sampling frame was the database of the anti-foot and mouth disease vaccination program, corresponding to the first period of 2018, which covered approximately 95% of the dairy cattle population in the area. According to this database, the dairy cattle population in the three municipalities in that year included 107,395 animals distributed in 8200 herds [26].

### Study design and sample size

In this cross-sectional probabilistic study, the sample size was calculated using the PROMESA™ Software version 2.3.0.2 (Massey University, New Zealand) by applying the “comparison of proportions” command. For this calculation, a confidence level of 95%, power of 90%, expected prevalence of *S. aureus* without the hypothesized risk factor (washing hands before milking each cow) (P1 = 0.1), and expected prevalence of *S. aureus* with the risk factor (not washing hands before milking each cow) (P2 = 0.45) [27] were considered. In addition, the multivariable analysis was adjusted, obtaining a total sample size of 108 herds. To improve the internal validity of the study, the sample size was increased to 150 herds.

### Herd selection

Considering the aforementioned sampling framework, stratified random sampling was carried out, where the number of herds sampled was proportional to the total number of cows in milk production per municipality. Herd selection was carried out in the three districts of each municipality with a higher number of cows in milk production. The number of herds selected for each district was proportional to their percentage share in the number of herds in the municipality.

In all cases, herds had to fulfill the following inclusion criteria: (1) Having a BTM – not shared or a community one; (2) willingness of the owner to participate (i.e., allowing the sampling and giving information indicated in the questionnaire) voluntarily; and (3) having an easily accessible herd.

For the random herd selection, a consecutive number was assigned to each herd in each selected district, and random numbers were then chosen using the “random number” tool of Microsoft Excel (Microsoft Corp., Redmond, WA, USA). Using the contact list, one of the authors contacted the herd owner or manager by phone, to validate compliance with the inclusion criteria and schedule the visit. When one of the herds must be replaced, the researchers selected another herd from the list of eligible ones.

### Herd visit protocol and samples collection

A single visit was made to each of the 150 herds, between August 7, 2019, and January 18, 2020. An epidemiological survey was conducted in each herd to identify herd-level and milking management practices. Bulk tank milk sampling was conducted in each herd once. Before the collection of the milk sample, the raw milk inside the bulk tank was shaken, and from each tank (equivalent to each herd), three milk samples were collected aseptically in 33 mL sterile screw-top plastic containers, following the protocols established by the National Mastitis Council of the United States [1]. The containers were labeled with a consecutive number from 1 to 150, herd data, and collection date. One of the containers contained the preservative azidiol to evaluate the SCC, whereas the other two that did not contain a preservative were used to obtain the samples for the microbiological isolation of *S. aureus*, *E. coli*, and *Klebsiella* spp. All samples were transported under refrigeration at 4°C in Styrofoam coolers with sealed ice blocks until they arrived at the Diagnostic Unit of the Faculty of Agrarian Sciences. Once at the laboratory, samples were kept under refrigeration for a maximum of 24 h until analysis.

### Laboratory analyses of samples

Each of the 150 BTM samples was left at room temperature (22°C) for 30 min and homogenized 30 times by inversion. Microbiological procedures were performed in a Type II Biosafety cabinet. Briefly, using a sterile calibrated loop (Biologix^®^ Group Ltd., Shandong, China), 0.01 mL of milk samples was streaked on Columbia agar plates supplemented with 5% sheep blood (BioMérieux, Marcy-l’Étoile, France). This procedure was repeated on MacConkey agar plates (Merck, Darmstadt, Germany). Each plate was incubated at 37°C for 48 h. Colonies on Columbia agar plates were selected by morphology, Gram stain, and catalase test result. Gram-positive and catalase-positive colonies were subjected to BD BBL^®^ tube coagulase (Becton Dickinson, Dublin, Ireland) and plate mannitol (Merck) biochemical tests. Isolates positive for all biochemical tests were selected as *S. aureus*. Colonies grown on MacConkey agar plates were subjected to the oxidase strip test (Merck). The oxidase-negative colonies were subjected to biochemical tube tests to screen for identification on the genus and species levels, triple sugar iron agar, indole, citrate, urea, and lysine.

### Somatic cell count determination

Each of the 150 BTM samples was analyzed by automated procedures using flow cytometry with the Fossomatic^®^ equipment (Fosss, Hillerød, Denmark).

### Case definition

A herd was defined as positive for any of the pathogens of interest (i.e., *S. aureus*, *E. coli*, *Klebsiella* spp.) if at least one isolate of the pathogen was found per milk sample. A herd was positive for Gram-negative bacteria when *E. coli* and/or *Klebsiella* spp. was isolated. For the SCC analysis, the cutoff SCC was 200,000 cells/mL, in which herds with SCC ≥200,000 cells/mL were considered affected by SM [28].

### Epidemiological survey

The questionnaires used in the identification of herd-level characteristics and milking management practices were validated before their application in the studied herds. The validation consisted of collecting information in a herd different from the study ones and the harmonization of the questions and concepts among all the interviewers, which was carried out to reduce the bias at the time of the visits in the study herds. The instrument was applied to the person in charge of milking at the time of the visit to the study herds. The research scope was verbally explained, and if the person of interest agreed to participate, a signed informed consent was obtained. The instrument is available on request to the corresponding author.

### Statistical analysis

All the information collected using the epidemiological survey and laboratory results were entered into Excel worksheets (Microsoft Corp.) and then exported to Stata 16.0^®^ (StataCorp, 2020, College Station, TX, USA) for statistical analysis. Data were examined for biologically implausible entries, and erroneous entries were removed or corrected. Descriptive statistics were calculated for all variables of interest. Variables with >30% missing values were eliminated. All study variables were described as frequency and percentage. For all study outcomes (i.e., positive herd for *S. aureus*, *E. coli*, and/or *Klebsiella* spp.), prevalence and a 95% confidence interval (95% confidence interval [CI]) were estimated and adjusted by sample design using Stata’s svy linearized command.

### Risk factor analysis

The crude prevalence ratio and its 95% CI municipality-adjusted cluster were estimated to determine the factors associated with each outcome, considering p < 0.05 as significant. In addition, Chi-square test, Fisher’s exact test, or the likelihood test was used to assess statistical significance, according to each case. For the SCC variable, the geometric mean and its 95% CI and statistical significance were estimated using the Mann–Whitney U test because the variables did not follow a normal distribution according to the Kolmogorov–Smirnov test. For the selection of the variables to be included in the multivariate models, those with p < 0.25 by the bivariate analysis and those previously reported in the literature were used. For dichotomous outcomes, binomial regression or Poisson regression was used when the former failed to converge. For continuous outcomes, a generalized linear regression model with γ-family and identity bond was used. All main exposures of interest were adjusted for potential confounding variables by readjusting the final models and omitting the variables to see if the coefficients of other predictors changed substantially. The interaction of variables was also evaluated by including the term in the model whenever it was significant. For the selection of the final model, Akaike’s information criterion was used. All analyzes were performed using Stata 16.0® software (StataCorp).

## Results

### Herd characteristics

In this study, 44.7% of the herds have <30 milking cows, half of the herds only have one milker, and a little more than 50% of the milkers own the herd (55.3%). In addition, according to herd type (i.e., cattle purchasing practices), 79.3% of the herds are closed. In the geographical area where the municipalities and herds are located, two milking times per day are common (98.7%), using milking mechanical devices (92%), and in-parlor milking facilities (45.3%). Therefore, most of the herds do not have automatic separators (82.7%); however, they conduct maintenance activities of the milking equipment (90%) at least every 6 months (57.4%) or every year (17.6%) (Table-1).

**Table-1:** Management characteristics of the 150 herds of study in three municipalities of Northern Antioquia, Colombia.

Variable	Category	Observations	Distribution (%)
Number of milking cows	<30	67	44.77
	30–60	64	42.7
	>60	19	12.7
Number of milkers	1	76	50.7
	2	66	44.0
	3	4	2.7
	4	4	2.7
The milker is the owner	Yes	83	55.3
	No	67	44.7
Number of milkings per day	1	1	0.7
	2	148	98.7
	3	1	0.7
Change of milker during the past month	No	118	78.7
	Yes	32	21.3
Predominant breed in the herd	Holstein	111	74
	Jersey	12	8
	Other^a^	27	18
Herd type (according to purchasing practices)	Open^b^	31	20.7
	Closed^c^	119	79.3
Milking system	Mechanical	138	92
	Manual	9	6
	Both	3	2
Milking location	In-paddock	81	54
	In-parlor	68	45.3
	Both	1	0.7
Automatic separators	Yes	15	10
	No	124	82.7
	Not applicable	11	7.3
Milking equipment maintenance (by a specialized technician)	No	6	4
	Si	135	90
	Not applicable	9	6
Frequency of milking equipment maintenance	Every 6 months	78	57.4
	Every year	24	17.6
	Other	34	25

a Includes Jerhol and crossbreeds.

b Allows the purchase of animals from other properties/cattle fairs any time of the year.

c Only allows the use of replacement animals from the same herd.

The disinfection of the milking system is conducted manually (64%) using a milking protocol (91.3%) on a formal basis (60%). For the milk tank, a little more than half of the herds (55.7%) use the formal disinfection protocol (Table-2). In relation to the adjustment of the milkers to the milking protocol, most producers (68.7%) disinfected their hands before milking, mainly with chlorinated (41.5%) and iodinated (19.0%) products. In addition, no milking gloves were used in 58.7% of the herds, and among those that used them, only 18.9% of the milkers disinfected themselves with chlorinated (64.3%) and iodinated (25%) products. Forestripping was conducted in 96.7% of the herds and pre-dipping in 81.3%, mainly with iodized products (69.7%), in a concentration recommended by the manufacturer (48.3%), for <15 s on the teat skin (51.7%). In 70.5% of the herds, individual teats were dried, mostly with newspaper (66.4%). For post-dipping (91.3%), iodinated products were used in 78.6% of the herds, in a concentration recommended by the manufacturer (88.5%) (Table-2).

**Table-2:** Cleaning, disinfection, and milking protocols used in the 150 herds of study in three municipalities of Northern Antioquia, Colombia.

Variable	Category	Observations	Distribution (%)
*Cleaning and disinfection protocols*			
Milking system disinfection	Automatic	45	30
	Manual	96	64
	Not applicable	9	6
Milking machine disinfection protocol	No	2	1.3
	Yes	137	91.3
	Not applicable	11	7.3
Protocol type	Formal^a^	84	60
	No formal^b^	55	39.3
	Not applicable	1	0.7
BTM disinfection protocol	Yes	149	99.3
	No	1	0.7
Type of BTM disinfection protocol	Formal^a^	83	55.7
	Not formal^b^	66	44.3
*Milking protocol*			
Hand disinfection before milking	Yes	103	68.7
	No	47	31.3
Hand disinfection product	Chlorinated	61	41.5
	Iodinated	28	19.0
	Other^c^	14	9.5
	Not applicable	44	29.9
Use of disposable gloves	Yes	62	41.3
	No	88	58.7
Hand disinfection during milking with gloves on	Yes	28	18.9
	No	118	79.7
	Not applicable	2	1.3
Product used to disinfect gloves	Chlorinated	18	64.3
	Iodinated	7	25
	Other^c^	3	10.7
Forestripping is performed	Yes	145	96.7
	No	5	3.3
Pre-dipping is performed	Yes	122	81.3
	No	28	18.7
Pre-dipping solution	Chlorinated	26	21.3
	Iodinated	85	69.7
	Other^c^	11	9
Pre-dipping product concentration	Recommended by the manufacturer	69	48.3
	Not recommended by the manufacturer	53	37.1
	Not applicable	21	14.7
Contact time of the teats with the pre-dipping product	<15 s	75	51.7
	15–30 s	23	15.9
	>30 s	24	16.5
	Not applicable	23	15.9
The pre-dipping product is dried	Yes	121	80.7
	No	1	7
	Not applicable	24	18.7
Teats are individually dried	Yes	105	70.5
	No	34	22.8
	Not applicable	10	6.7
Materials used in drying the teats	Rag	4	2.68
	Newspaper	99	66.4
	Paper towels	34	23.5
	Not applicable	11	7.4
Post-dipping is performed	Yes	137	91.3
	No	12	8
	Not applicable	1	0.7
Post-dipping solution	Chlorinated	9	6
	Iodinated	118	78.6
	Not applicable	13	8.7
	Other^c^	10	6.7
Post-dipping product concentration	Recommended by the manufacturer	131	88.5
	Not recommended by the manufacturer	6	4.1
	Not applicable	11	7.4

a Delivered by a specialized company.

b Own elaboration.

c Alcohol, chlorhexidine, and lactic acid. BTM=Bulk tank milk

### Prevalence of *S. aureus, E. coli,* and *Klebsiella* spp.

The microbiological culture of the 150 BTM samples showed an apparent prevalence for the three municipalities of 14% (21/150), 2% (3/150), and 8% (12/150) for *S. aureus*, *E. coli*, and *Klebsiella* spp., respectively. Moreover, 22.7% of the herds were positive for at least one of the pathogens analyzed, and 10.7% were positive for one of the coliform bacteria (*E. coli* and/or *Klebsiella* spp.).

### Somatic cell count

The analysis of the SCC of the BTM samples showed that in the 150 herds visited, a geometric mean of 445,200 (95% CI: 409,600–483,900) cells/mL and an arithmetic mean of 507,966 (95% CI: 464,380–551,552) cells/mL were found. Moreover, 94.9% (95% CI: 89.3–97.6) of the herds presented an SCC of ≥200,000 cells/mL.

### Risk factors associated with *S. aureus* isolation

According to the multivariate logistic regression analysis, in-paddock milking increases the prevalence of *S. aureus* isolation in BTM by 33%, compared with in-parlor milking. The change of milker during the last month tended to be associated with a 50% increase in the prevalence of *S. aureus* compared with those who did not report such change (p = 0.08). Hand disinfection between cows, even when gloves are used, decreased the prevalence of *S. aureus* by 92% compared with not doing so. The use of chlorinated or other disinfectants (i.e., alcohol, chlorhexidine, and lactic acid) as hand disinfection products was associated with a 40% and 43% lower prevalence of *S. aureus*, compared with not disinfecting hands. Herds that used disposable gloves tended to have increased *S. aureus* incidence by 2.2 times compared with herds that did not use them (p = 0.07). Appropriate teat post-dipping decreases the prevalence of *S. aureus* by 49% compared with not doing so. An interaction effect was observed between the number of milking cows and hand disinfection between cows, which increased the prevalence of *S. aureus* in BTM samples by 3.5 times (Table-3).

**Table-3:** Risk factors associated with the isolation of *S. aureus* of the 150 herds of study in three municipalities of Northern Antioquia, Colombia.

Variable	Category	Risk ratio	Standard error	p-value	95% CI
Number of milking cows	30–60	0.80	0.53	0.74	0.22–2.91
	>60	0.44	0.36	0.32	0.87–2.21
Breed	Holstein	1.57	0.77	0.35	0.60–4.08
	Jersey	0.72	0.89	0.79	0.06–8.17
The milker is the owner		1.78	1.70	0.54	0.27–11.60
Milking system		1.15	1.10	0.88	0.18–7.58
Milking location	In-paddock	1.33	0.09	**0.00**	1.16–1.52
Change of milker during the last month		1.50	0.35	0.08	0.94–2.37
Herd type (according to purchasing practices)		1.39	0.51	0.36	0.67–2.87
Hand disinfection between cows		0.08	0.06	**0.00**	0.02–0.40
Hand disinfection product	Chlorinated	0.60	0.11	**0.00**	0.42–0.86
	Iodinated	0.99	0.60	0.98	0.30–3.26
	Other^a^	0.57	0.11	**0.00**	0.40–0.83
Use of disposable gloves		2.26	1.03	0.07	0.93–5.53
Proper teat post-dipping		0.51	0.10	**0.00**	0.35–0.75
Cows in milking and hand disinfection between cows*		3.5	1.83	**0.018**	1.24–9.76

* Result of the interaction between the mentioned variables.

a Alcohol, chlorhexidine, and lactic acid. CI=Confidence interval, Bold numbers represent significant association (p < 0.05).

### Risk factors associated with *E. coli* and *Klebsiella* spp. isolation

According to the multivariable logistic regression analysis, large herds (i.e., >60 milking cows) were associated with a 3.48-fold increase in the prevalence of *E. coli* and *Klebsiella* spp. isolation, compared with herds with <30 milking cows. The change of milker during the last month was associated with an 83% increase in prevalence compared with those who did not change. Appropriate washing and disinfection of the milking machine were associated with a 92% decrease in the prevalence. The prevalence decreased by 44% in herds that used disposable gloves compared with those that did not. Hand disinfection between cows, even when gloves were on, increases the prevalence by 2.61 times compared with not doing it. The use of chlorinated disinfectants for hand hygiene was associated with a 91% lower prevalence than not sanitizing hands. The use of iodized and other pre-dipping products reduced the prevalence by 80 and 98%, respectively, compared with not doing so. The use of the manufacturer’s recommended pre-dipping product concentration was associated with a 23.7-fold increase in the prevalence compared with not pre-dipping. A contact time of the teats’ skin with the pre-dipping solution >30 s was associated with a 94% decrease in the prevalence. The interaction between the change of milker during the last month and appropriate washing and disinfection of the milking machine was associated with a 7.28-fold increase in the prevalence of both coliform bacteria (Table-4).

**Table-4:** Risk factors associated with the isolation of *E. coli* and *Klebsiella* spp. of the 150 herds of study in three municipalities of Northern Antioquia, Colombia.

Variable	Category	Risk ratio	Standard error	p-value	95% CI
Number of milking cows	30–60	0.95	0.23	0.84	0.58–1.54
	>60	3.48	0.62	**0.00**	2.45–4.95
Change of milker during the last month		1.83	0.40	**0.00**	1.19–2.82
The milker is the owner		3.44	2.91	0.14	0.65–18
Milking system		0.57	0.59	0.59	0.07–4.27
Milking location		0.57	0.17	0.07	0.32–1.03
Proper washing and disinfection of the milking machine		0.076	0.016	**0.00**	0.050–0.11
Proper disinfection of the milk tank		0.88	0.43	0.80	0.34–2.27
Use of disposable gloves		0.56	0.14	**0.01**	0.35–0.91
Disinfection of hands wearing gloves		2.61	0.5	**0.00**	1.79–3.79
Hand disinfection product	Chlorinated	0.087	0.077	**0.00**	0.01–0.49
	Iodinated	0.20	0.22	0.13	0.02–1.63
	Other^a^	0.64	0.30	0.36	0.25–1.64
Pre-dipping product	Chlorinated	0.47	0.24	0.14	0.17–1.29
	Iodinated	0.20	0.11	**0.00**	0.06–0.59
	Other^a^	0.02	0.01	**0.00**	0.01–0.06
Use of manufacturer’s recommended pre-dipping product concentration		23.7	14.11	**0.00**	7.39–76
Contact time of the teats’ skin with the pre-dipping solution	15–30 s	0.87	0.38	0.76	0.36–2.07
	>30 s	0.06	0.05	**0.00**	0.01–0.30
Change of milker during the last month and proper washing and disinfection of the milking machine*		7.28	5.12	**0.00**	0.01–0.30

* Result of the interaction between the mentioned variables.

a Alcohol, chlorhexidine, and lactic acid. CI=Confidence interval, Bold numbers represent significant association (p < 0.05).

### Risk factors associated with the SCC

According to the generalized linear regression analysis, the SCC increases as the number of milking cows also does; thus, the SCC increased by an average of 117,000 cells/mL in the BTM of herds with 30–60 milking cows compared with those with <30 milking cows, whereas herds with >60 milking cows had an average increase of 155,000 cells/mL. The SCC decreased by 35,000 cells/mL on average in herds that had a change of milker during the last month compared with those that did not change. Compared with herds that did not use hand disinfection, those that used other disinfectants (i.e., alcohol, chlorhexidine, and lactic acid) as hand disinfection products showed a trend of increasing SCC by 176,000 cells/mL on average. Hand disinfection between cows was associated with a decrease of 150,000 cells/mL on average compared with not doing this procedure within the milking routine. Appropriate fore-stripping was associated with a decrease of 116,000 cells/mL on average in the BTM compared with those that did not undergo this procedure (Table-5).

**Table-5:** Risk factors associated with the somatic cell count of the 150 herds of study in three municipalities of Northern Antioquia, Colombia.

variable	Category	Risk ratio	Standard error	p-value	95% CI
Number of milking cows	30–60	116.92	37.54	**0.00**	43.33–190.52
	>60	154.73	61.37	**0.01**	34.43–275.02
Breed	Holstein	16.44	47.59	0.73	−76.84–109.73
	Jersey	−20.21	96.72	0.83	−209.78–169.36
Herd type (according to purchasing practices)		125.32	101.68	0.218	−73.97–324.63
The milker is the owner		36.35	54.50	0.50	−70.47–143.17
Change of milker during the last month		−35.59	11.54	**0.00**	−58.22–12.97
Milking system		−83.75	398.21	0.83	−864.23–696.72
Milking location		23.11	24.08	0.33	−24.08–70.31
Automatic separators		10.02	70.08	0.88	−127.34–147.40
Disinfection product	Chlorinated	30.53	65.08	0.63	−97.04–158.10
	Iodinated	52.89	113.50	0.64	−169.56–275.35
	Other^a^	176.03	91.72	0.06	−3.75–355.81
Use of disposable gloves		−32.51	25.70	0.20	−82.89–17.86
Hand disinfection between cows		−150.04	58.25	**0.01**	−264.21–−35.87
Forestripping		−116.49	52.40	**0.02**	−219.20–−13.79
Pre-dipping		−15.00	67.58	0.82	−147.48–117.46
Contact time of the teats’ skin with the pre-dipping solution	15–30 s	−25.14	50.72	0.62	−124.56–74.28
	>30 s	3.65	26.66	0.89	−48.61–55.92
Proper post-dipping		15.83	121.86	0.89	−223.01–254.68

*Result of the interaction between the mentioned variables.

a Alcohol, chlorhexidine, and lactic acid. CI=Confidence interval, Bold numbers represent significant association (p < 0.05).

## Discussion

This study was conducted in specialized dairy herds located in one of the main dairy basins in Colombia, and since the country’s specialized dairy areas share similar characteristics, such as average temperature, specialized dairy breeds (Holstein and crosses with a local breed, American–Swiss–Brown, and Jersey), development feeding systems, and infrastructure, focused on milk production [29], two milkings per day, and supplementation with concentrated feed during the milking, our results could be representative of the characteristics, pathogen situation, and risk factors of this product in the country.

In specialized dairy systems in Colombia, the cows are not stable, and the cows are fed directly on pasture grass (*Crencus clandestinum*) and are only supplemented at the milking time with commercial concentrate. Conversely, Colombia is not a seasonal country, and its climate varies between dry and rainy periods; therefore, pastures are present throughout the year. For all of the above and because this study did not evaluate individual animal conditions, the authors did not consider the effect of feeding on the results of this study; thus, it could be taken into account for future research.

Regarding the characterization of the herds studied, the results of this study are consistent with others in the region, in which an average of 25.6, 32, and 49 milking cows were identified [21, 25, 30]. This reveals that milk production is related to small- and medium-scale producers with low resources, which reinforces the need for greater technical support from the dairy industry, government, and universities in the study area.

The finding that more than half of the milkers were the owners of the cows differs from previous reports in the country [31], in which only 6% of the people, who carried out this work, were the owners of the animals. In this sense, an increase in *S. aureus*-related cases has been identified in herds in which the milkers are not the owners of the animals; thus, it is necessary to train employees frequently, review milking procedures, and correctly implement them to maintain good udder health [32, 33]. This was further confirmed by the finding of this study, where changing milking personnel intermittently (change of milker during the last month) is a risk factor for the emergence of *S. aureus*.

Contrastingly, mechanical milking has been implemented by most of the study herds (92%), which, according to some authors, indicates technification or intensification of the production system; therefore, these systems have allowed improving parameters related to milk production and proportion of milking cows [30]. In relation to the milking system, although manual milking was found less frequently and has decreased compared with the previous reports of other authors [21, 25], this system is still in force in the area and is a risk factor for the presentation of contagious pathogens such as *S. agalactiae* [21], which has transmission and dissemination similar to *S. aureus*.

The prevalence of *S. aureus* in this study is lower than that reported by the previous studies conducted in BTMs in Colombia [34, 35] and by other countries such as Brazil [36], United States [37], Canada [38], and Denmark [39], but higher than that reported by previous studies conducted in England [40]. The differences in prevalence may be due to the geographical particularities of each region, with heterogeneous agroecological, technological, and socioeconomic conditions [29] and their association with management changes, and control programs implemented in these geographical areas.

*Staphylococcus aureus* isolation in BTM indicated that milking cows were infected with this pathogen inside the herds [11]. This is important because *S. aureus* successfully remains in the cow population and it can be spread by the milker’s hands or milking machine from the infected animals to healthy ones during milking. In addition, its virulence factors allow it to establish itself subclinically and chronically in the mammary gland, turning infected animals into primary reservoirs and increasing the risk of intra-herd spread [2, 41]. Therefore, eliminating chronically affected animals and milking infected cows in a separate group or at the end of milking [42], combined with the establishment of a comprehensive control program aimed to reduce the occurrence of pathogens [2], was proposed to reduce the reservoir in herds.

The presence of *S. aureus* in the study herds could be due to their herd type (according to purchasing practices) *—* where 21% of the herds are open—and lack of effective prevention, control, and eradication plans in the area, coupled with the little or scarce implementation of diagnostic tools to identify the organism with the highest incidence in animals. In this way, the presence of infected animals in the herd without a clear diagnosis of the same, and therefore without actions aimed at its control, increases the risk of dissemination to healthy animals, people who consume raw milk, or those who are in direct contact with the animals. Consequently, implementing udder health programs complemented with direct educational campaigns to milkers and workers is necessary to correct the problems [31].

The prevalence of *E. coli* found in this study is lower than that reported by the previous studies conducted in BTM in the country (*E. coli*, 68.75%; *Klebsiella* spp., 5%) [34, 35]. However, higher prevalence rates have been reported in other countries such as the United States (3.8%) [43], Korea (63.1%) [44], and Brazil (74.55%) [45]. The differences in the prevalence within the country could be related to the differences in the production and milking systems, which vary between those located in the low tropics compared with the systems implemented in the specialized dairy, usually in the high tropics. The differences between the prevalence among countries could be due to dairy cattle management changes and modernization present in more industrialized countries with stable production systems, compared with Colombia.

A higher prevalence of *Klebsiella* spp. was found when compared with *E. coli* in the study BTMs. *Klebsiella* spp. is found in the environments, especially in used bedding material, fecal shedding, soil, water troughs, rumen content, alleyways, holding pens, and water [17, 46]. Because the herds in this study are based on grazing systems, that is, there is no confinement, the authors hypothesize that the increased prevalence of *Klebsiella* spp. may be due to washing utensils with running water (contaminated with the pathogen) in the study herds; therefore, the recommendation is to improve the hygienic quality of water for washing utensils and milking practices to avoid contamination of pathogens that can affect people who consume raw milk or its derivatives [45].

Although this study cannot guarantee that the isolated Gram-negative bacteria come from the animals themselves, bacteria such as *E. coli* could contaminate the milk during or between milkings, thus reaching the BTM [11]. In addition, these bacteria could come directly from the udders of cows that may be affected by SM. However, with the type of samples obtained herein, the origin of these bacteria cannot be defined. This aspect is one of the limitations of this study.

The contamination of the BTM by coliform bacteria such as *E. coli* and *Klebsiella* spp. is a matter of concern in recent times because *Enterobacteriaceae* have been reported to have virulence factors that can cause hemorrhagic syndromes or antimicrobial resistance factors in strains isolated from raw milk or its derivatives, representing a risk to public health. In this sense, establishing control and prevention measures is necessary, especially in the milking routine and procedures, to prevent their presentation in the cooling tank [47], preventing risks to humans, especially in a susceptible population such as children who consumed this food in a greater quantity.

The average SCC in the study herds was lower than that found in BTM in the province of Valle del Cauca (Colombia) and the same region of the present study [25, 34], but higher than that found in dual-purpose systems in the province of Córdoba (Colombia) [18]. Globally, it was also higher than that in studies conducted in Tunisia [9] and Canada [38]. The possible reason for the differences between regions is having areas with the least SCC have dual-purpose systems, located in low-lying tropical areas (<1200 m.a.s.l.), where *Bos indicus* and *Bos taurus* crossbreeds predominate, the cows are milked once a day, and the calf remains next to the mother during milking and grazing [48]. The foregoing reflects that the milking routine varies according to the conditions of the production systems, which could influence the incidence of pathogens in the mammary gland and therefore, the value of the SCC in the BTM.

The findings of this study show that most of the herds analyzed had an SCC of >200,000 cells/mL, a cutoff point that has been used in other countries for monitoring milk quality in udder health programs, both in animals and BTM [38]. According to this, most of the study herds have animals affected with SM [28] and are probably infected with *S. aureus*.

In the multivariate analysis, inadequate practices during milking increased the risk of intramammary infections by *S. aureus*, which therefore, can increase the SCC in the quarter and finally in the BTM. Thus, the findings at this level reinforce the concept that BTM SCC assessment is an inexpensive diagnostic tool that assists veterinarians and producers in monitoring mastitis-specific pathogens in a cow population [49] since a highly meaningful relationship has been known between the number of contaminations with mastitis pathogens and the increase in SCC in BTM [50]. Thus, implementing this diagnosis in control and monitoring programs in herds in tropical countries such as Colombia was proposed.

Given that the SCC increases as the size of the herd does, this could be due to a greater control applied over the processes, such as the milking protocol, for the prevention of contagious pathogens in small herds. This finding is consistent with those of other studies in which a higher prevalence of *S. aureus* (47%) and a higher SCC were found in large herds [11, 22, 38]. In Colombia, there are no government regulations for mandatory bonuses or penalties according to the result of the SCC in BTM, and these types of incentives or penalties are applied voluntarily by the industry. The SCC bonus promotes control and a decrease in its values in BTM compared with those that are not subsidized [11], putting pressure on producers to improve the sanitary quality of raw milk and the implementation of bovine mastitis prevention and control programs.

Interestingly, in this study, the in-paddock milking system was a risk factor for *S. aureus* isolation in the herd. In this study, 88% of the in-paddock milking systems were mechanical, which probably means that this special characteristic of this system (mechanical in-paddock) in the region is a risk factor for the infection of cows with *S. aureus*. However, more studies are needed to understand this possible link. Although the milking system characteristics are not the same, these findings are consistent with a study that found a higher probability of isolating the pathogen in herds that used mobile milking machines in Portugal [51]. At least in Colombia, this could be related to the variability of the pastures and paddock conditions that can be affected by rainfall and thus influence the incidence of pathogens and, therefore, SCC [25]. In addition, the difficulties involved in disinfecting mobile equipment and in-paddock milking that are outdoors make them more prone to contamination and spread of contagious pathogens. The mechanical milking factor in in-paddock locations could be a target of future research aimed at knowing its relationship with the presentation of mastitis and a high SCC in dairy herds.

The milking protocol is part of the internationally established control and prevention measures such as the 10-step program to prevent and control bovine mastitis [2, 3]. This study was based on such a protocol to evaluate the execution of the established measures, finding interesting associations related to *S. aureus* positivity in BTM. Among them, surprisingly, the use of disposable gloves showed a trend to increase the prevalence of pathogens. Although disposable gloves did not show statistical significance, the trend is noteworthy given that this finding differs from what was evaluated in other studies, in which the use of disposable gloves decreases the incidence of *S. aureus* and other contagious pathogens. In addition, not wearing gloves was related to herds with BTM SCC ≥200,000 cells/mL [47, 52]. Since the used disposable gloves, without washing or disinfecting between animals, can become a fomite and disseminator of pathogens in herds infected with contagious bacteria, transmission of the agent occurs from an infected animal to a healthy one during the milking process [2]. This is consistent with the finding in this study, where hand disinfection between cows and the use of disinfectants for hand hygiene, even when gloves were on, was a protective factor for the presentation of the agent, that is, the role that disinfection played was even more important than the physical barrier of the gloves.

Adequate post-dipping of teats was a protective factor for *S. aureus* isolation, which agrees with the findings of other studies that not executing it was a risk factor for the presentation of the agent and SM in cows [8, 31, 53].

Most of the producers in the evaluated area follow the classic milking protocol (i.e., forestripping, pre-dipping, individual drying of teats with paper, and post-dipping). However, practices such as drying teats with newspaper, which is frequently used in dairies in the country, despite not being supported by any study, are recommended by technicians and professionals in the sector.

Although most herds followed the routine milking steps, it is necessary to harmonize their execution protocol in the herds, because, according to the study finding, details in the implementation of this protocol can interfere with the effective prevention and control of milking pathogens. A study identified that the implementation of standardized, systematic, and constant programs for the prevention of mastitis have reduced the SCC in BTM samples and the prevalence of pathogens such as *S. aureus* [31].

We confirmed that some processes (i.e., proper washing and disinfection of the milking machine, use of chlorinated disinfectants for hand hygiene, and use of disposable gloves) decrease the prevalence of Gram-negative pathogens (*E. coli* and *Klebsiella* spp.) in raw milk. These findings are consistent with those of other studies in which increased bacteria counts in milk were associated with poor milking hygiene, dirty equipment, inadequate disinfection practices, and milk stone deposits in pipes, suggesting that an inadequate cleaning system can be an important risk factor for BTM contamination [54]. Therefore, appropriate implementation of these practices decreases the bacterial load of the water used to clean the milking equipment and hand washing as a potential source of bacterial contamination [55].

This study showed that hand disinfection between cows *—* even when gloves are used — increased the prevalence of *E. coli* and *Klebsiella* spp. in BTM. However, given that these bacteria have an environmental origin and they do not survive long on the skin [17, 46], this finding could confirm that environmental pathogens come from the external contamination to the milker’s hands and confirms the importance of controlling environmental sources of contamination, which may be because most of the study herds conduct manual disinfection of the milking equipment and some producers follow equipment washing protocol empirically.

The isolation of Gram-negative bacteria has also been associated with poor hygiene in the environment where the animals stay and absence of hygienic milking practices, such as pre-dipping [21, 55]. In this study, pre-dipping with iodinated products for 30 s reduces the prevalence of *E. coli* and *Klebsiella* spp. in BTM because the use of disinfectants before milking helps reduce the bacterial load on the skin of the teats that could enter the mammary gland and raw milk [55]. This reduces the potential risk for the establishment of new intramammary coliform infections and a source of this type of pathogen for the human population that consumes raw milk or cheese [31].

To our surprise, the use of the manufacturer’s recommended pre-dipping concentration was associated with an increased prevalence of *E. coli* and *Klebsiella* spp. This result is consistent with the findings of other authors who performed a meta-analysis, revealing that the concentration of the active ingredient may not be a good predictor of the efficacy of the product because other factors (such as free available iodine) influence the efficacy of pre-dipping [56].

In this study, the increase in the number of milking cows was associated with an increase in the probability of isolating *S. aureus*, *E. coli*, and *Klebsiella* spp. and the increase in SCC in the BTM. This finding agrees with the results of other studies in that SCC, and consequently mastitis, increased significantly with herd size [11, 57].

A lower SCC was found in herds that performed forestripping. This has been previously identified by other authors who reported that forestripping is one of the best methods to reduce SCC in the BTM, being also a method that allows the identification of changes in milk appearance and animals affected by clinical mastitis and avoids adding low/poor quality milk to the bulk tank [57].

Inadequate hygiene practices can increase the probability of isolating *S. aureus* and consequently increase the SCC in the BTM [25, 51], which is consistent with our findings, where performing an adequate milking protocol allowed for reducing the average SCC in herds. In this study, the practices that were found to be associated with the decrease in SCC were the use of other hand disinfection products (i.e., alcohol, chlorhexidine, and lactic acid), hand disinfection between cows, and forestripping.

Importantly, Colombia does not have a national SCC control and evaluation program; therefore, the implementation of regional or national mastitis monitoring programs is recommended, where SCC is evaluated at least once a month to give special attention to chronic cases, determine the order of entry to milking, and make decisions to dispose of infected animals [31].

Conducting a single visit per herd and obtaining data directly from the milkers, not from other sources, are limitations of this study because data provided could bias the reality of what is implemented in milking. Despite this, producers provided all the requested information without difficulty.

## Conclusion

This study highlights the importance of identifying the risk factors present in developing tropical countries such as Colombia, whose milking systems are diverse and constantly changing (manual milking to the mechanical parlor or paddock systems). According to the results, *S. aureus*, *E. coli*, and *Klebsiella* spp. were prevalent in BTM from dairy cow herds. The risk of *S. aureus* isolation was higher in herds with an in-paddock milking system, probably in-paddock mechanical milking; however, more studies are needed to understand this possible link. The risk of *E. coli* and *Klebsiella* spp. isolation was higher in herds with >60 milking cows, with a change of milker during the past month, and when hand disinfection between cows is carried out, even if gloves are on. Processes such as avoiding changing the milker and greater control in medium and large herds could improve the SCC in BTM. Finally, implementing a national udder health monitoring program, considering the conditions of the regions, is necessary to influence the practices that can alter the quality of raw milk and the health of cows.

## Authors’ Contributions

JAF and NFR: Designed the study. ASA: Collected samples, performed experiments and drafted the manuscript. JAF, NFR, and ASA: Analyzed and interpreted the data. All authors have read, reviewed, and approved the final manuscript.
